# c-Fms Signaling Mediates Neurofibromatosis Type-1 Osteoclast Gain-In-Functions

**DOI:** 10.1371/journal.pone.0046900

**Published:** 2012-11-07

**Authors:** Yongzheng He, Steven D. Rhodes, Shi Chen, Xiaohua Wu, Jin Yuan, Xianlin Yang, Li Jiang, Xianqi Li, Naoyuki Takahashi, Mingjiang Xu, Khalid S. Mohammad, Theresa A. Guise, Feng-Chun Yang

**Affiliations:** 1 Department of Pediatrics, Indiana University School of Medicine, Indianapolis, Indiana, United States of America; 2 Department of Anatomy and Cell Biology, Indiana University School of Medicine, Indianapolis, Indiana, United States of America; 3 Division of Hard Tissue Research, Matsumoto Dental University, Shiojiri, Nagano, Japan; 4 Department of Medicine, Indiana University School of Medicine, Indianapolis, Indiana, United States of America; University of Toronto, Canada

## Abstract

Skeletal abnormalities including osteoporosis and osteopenia occur frequently in both pediatric and adult neurofibromatosis type 1 (NF1) patients. *NF1* (*Nf1*) haploinsufficient osteoclasts and osteoclast progenitors derived from both NF1 patients and *Nf1^+/−^* mice exhibit increased differentiation, migration, and bone resorptive capacity *in vitro*, mediated by hyperactivation of p21^Ras^ in response to limiting concentrations of macrophage-colony stimulating factor (M-CSF). Here, we show that M-CSF binding to its receptor, c-Fms, results in increased c-Fms activation in *Nf1^+/^* ^−^ osteoclast progenitors, mediating multiple gain-in-functions through the downstream effectors Erk1/2 and p90RSK. PLX3397, a potent and selective c-Fms inhibitor, attenuated M-CSF mediated *Nf1^+/−^* osteoclast migration by 50%, adhesion by 70%, and pit formation by 60%. *In vivo*, we administered PLX3397 to *Nf1* ^+/*−*^ osteoporotic mice induced by ovariectomy (OVX) and evaluated changes in bone mass and skeletal architecture. We found that PLX3397 prevented bone loss in *Nf1^+/−^*-OVX mice by reducing osteoclast differentiation and bone resorptive activity *in vivo*. Collectively, these results implicate the M-CSF/c-Fms signaling axis as a critical pathway underlying the aberrant functioning of *Nf1* haploinsufficient osteoclasts and may provide a potential therapeutic target for treating NF1 associated osteoporosis and osteopenia.

## Introduction

Neurofibromatosis type 1 (NF1) is one of the most common genetic diseases with an incidence of 1 out of 3000 individuals [1]. NF1 is caused by mutations of the *NF1* tumor suppressor gene, which encodes neurofibromin, a GTPase activating protein for p21ras [2]. Individuals with NF1 have a high incidence of both malignant and non-malignant complications [3], [4]. Clinical studies have reported that NF1 patients are at a significant risk for both generalized osteoporotic abnormalities [5], [6], [7] and focal skeletal abnormalities including dystrophic kyphoscoliosis and pseudarthrosis [8], [9]. NF1 patients have an increased prevalence of osteoporosis beginning from childhood and adolescence [10], [11], [12], leading to greater risk of fracture later in life [13]. Given that osteoporosis occurs in a younger patient population and the predisposition to pseudarthrosis is 2–5% in individuals with NF1 [3], [14], [15], the ultimate health costs and sequelae of this condition in NF1 patients may be significantly greater. Although NF1 related osteopenia commonly presents in the childhood years, there is no efficient treatment so far. Despite evidence of low serum Vitamin D levels in some NF1 patients, clinical studies involving Vitamin D supplementation have yielded conflicting results on whether improvements in bone mineral density (BMD) can be achieved [10], [16], [17].

Osteoclasts are specialized bone resorbing cells which differentiate from the myeloid monocyte/macrophage lineage. Many skeletal diseases, in particular diseases with decreased bone mineral density (BMD), occur as a consequence of a skeletal imbalance that favors bone resorption. Although a significant number of skeletal diseases, including skeletal manifestations in NF1 patients, have been linked to abnormal osteoclast function(s) [18], [19], [20], [21], the intracellular mechanisms by which osteoclasts normally function or contribute to disease states are poorly understood.

Ras signaling pathways are highly relevant to bone formation and the maintenance of skeletal homeostasis. Several Ras-activating growth factors, including M-CSF, are known to affect skeletal development and remodeling. Mitogen-activated protein kinase, a major downstream effector of Ras, is critical in the mitogenic response to extracellular stimuli including growth, podosome formation, and bone resorption of the osteoclast [22]. Previously, we reported that haploinsufficient *Nf1* (^+/−^) myeloid progenitors are hypersensitive to M-CSF, leading to increased osteoclast formation and bone erosive activity *in vitro* [18]. Furthermore, we have shown that the phenotype of *Nf1^+/−^* osteoclasts appears to be associated with hyperactivation of the MAPK pathway [18].

Upstream of the Ras/MAPK pathway, M-CSF binding to its membrane receptor, c-Fms, stimulates phosphorylation of Y807 in the activation loop, resulting in a conformational shift that enhances intrinsic kinase activity and docking of adaptor proteins such as Grb2 and Sos with subsequent activation of the Ras/Raf/MEK/ERK cascade. Here we show that *Nf1* haploinsufficient osteoclast progenitors exhibit increased c-Fms activation in response to M-CSF, resulting in multiple osteoclast gain-in-functions including migration, adhesion, and bone resorptive capacity, which are correlated with hyperphosphorylation of the downstream effectors Erk1/2 and p90^rsk^. Administration of PLX3397, a potent and selective small molecule inhibitor of c-Fms receptor tyrosine kinase activity, was sufficient to mitigate hyperfunctioning *Nf1^+/−^* osteoclast phenotypes *in vitro*. *In vivo*, ovariectomized (OVX) *Nf1^+/−^* mice exhibit accelerated bone loss as compared to WT controls in response to bone resorptive stress. In the present study, we found that treatment with PLX3397 was sufficient to correct this phenotype, normalizing bone mineral density and trabecular bone mass in *Nf1^+/−^* -OVX mice vs. vehicle treated controls. Collectively, these data implicate the M-CSF/c-Fms signaling axis as a critical pathway underlying the aberrant functioning of *Nf1* haploinsufficient osteoclasts and warrant further investigation of c-Fms as a potential therapeutic target for treating NF1 associated osteoporosis and osteopenia.

## Materials and Methods

### Ethics Statement

This study was carried out in strict accordance Indiana University's Institutional Animal Care and Use Committee (IACUC). Animals and records of their ovariectomy surgery were maintained in compliance with Indiana University's Institutional Animal Care and Use Committee with approval protocol ID #3401- A4. All surgery was performed under isoflurane anesthesia, and all efforts were made to minimize suffering.

### Animal and material preparation

*Nf1^+/−^* mice were obtained from Tyler Jacks at the Massachusetts Institute of Technology (Cambridge, Massachusetts, USA) in a C57BL/6J.129 background and backcrossed for 13 generations into a C57BL/6J strain [23]. Studies were conducted with a protocol approved by the Indiana University Laboratory Animal Research Center. Chemicals were purchased from Sigma (St. Louis, MO) unless otherwise stated. All cytokines were purchased from PeproTech (Rocky Hills, NC). PLX3397 was provided by Plexxikon Inc. (Berkeley, CA).

### Clonogenic progenitor assays

Colony-forming unit-macrophage/monocyte (CFU-M) of bone marrow mononuclear cells (BMMNCs) was assayed, as described previously [19]. BMMNCs were isolated from 6- to 8-week-old WT and *Nf1^+/−^* mice by flushing the bone marrow and using Ficoll density gradient centrifugation. 2.5×10^4^ BMMNCs were seeded onto a 35-mm gridded dish containing methylcellulose supplemented with 30 ng/mL M-CSF and 20 ng/mL murine recombinant receptor activator of nuclear factor kappa-B ligand (RANKL) at 37°C in a 5% CO_2_ incubator for 7 days. Colonies were counted using an inverted light microscope.

### Generation of murine osteoclasts

Murine osteoclasts were obtained *in vitro* from BMMNCs, as described previously [18]. BMMNCs were cultured in alpha minimum essential medium (α-MEM) supplemented with 10% fetal bovine serum (FBS, Sigma) and M-CSF 30 ng/mL and RANKL 20 ng/mL for 3 days. Cell culture media was switched on day 4 to α-MEM supplemented with 10% FBS, M-CSF (30 ng/mL), and RANKL (60 ng/mL) for an additional 3 days of culture. Adherent cells were fixed and stained for tartrate resistant acid phosphatase (TRACP) to identify osteoclasts, as described previously [18]. Osteoclasts were visualized using a Nikon TE2000-S microscope (Nikon Inc., Melville, NY) and images were taken by a QImaging camera and QCapture-Pro software (Fryer Company Inc., Cincinnati, OH). Multinucleated TRACP^+^ cells containing more than three nuclei were scored as mature osteoclasts. The numbers and areas of multinucleated, TRACP^+^ osteoclasts were calculated using Image Pro Express (Media Cybernetics, In., MD).

### Migration assay

Osteoclast migration was evaluated using a transwell assay, as described previously with minor modifications [18]. Briefly, equivalent numbers of pre-osteoclasts were loaded onto the upper chamber of an 8 µm polycarbonate transwell (Corning Incorporated, Corning, NY) coated with vitronectin (Takara, Japan). The lower chamber contained α-MEM supplemented with M-CSF (30 ng/mL) and 0.1% bovine serum albumin with phosphate buffered saline (PBS) vehicle or PLX3397 (200 nM). Cells that migrated to the bottom chamber were stained with crystal violet and counted. Six fields per experimental condition were counted.

### Adhesion assay

A single-cell suspension of osteoclast precursors were pipetted into 96-well plates pre-coated with vitronectin at a density of 1×10^4^ cells/well as previously described [19]. After 30 minutes of incubation in the presence of M-CSF (30 ng/mL) with PBS vehicle or PLX3397 (200 nM), nonattached cells were gently removed with PBS and adherent cells were stained with crystal violet and counted.

### Bone resorption assay

Osteoclasts can form a specialized cell-extracellular matrix to initiate degradation of bone matrix by secreting proteinases. To directly assess the effect of PLX3397 on osteoclast bone resorption, independent of adhesion and differentiation, pit formation assay was performed using cells that were cultured for 3 days in media containing M-CSF and RANKL as described previously [18]. Briefly, preosteoclasts were detached from plates after 3 days culture in α-MEM supplemented with M-CSF (30 ng/mL) and RANKL (20 ng/mL) and replated at 1×10^5^/well on dentine discs (ALPCO Diagnostic, Windham, NH) in a 96-well plate. The cells were incubated at 37°C, 5% CO_2_ in the presence of 30 ng/mL M-CSF and 60 ng/mL RANKL and allowed to adhere overnight. On the following day, vehicle (PBS) or PLX3397 (200 nM) was added to the culture with media changed every three days. Wells containing media alone, without M-CSF, were used as negative controls (data not shown). Following 7 days of culture, the dentine discs were rinsed with PBS, immersed in 1 M ammonium hydroxide overnight, and stained with a 1% toluidine blue in 0.5% sodium tetraborate solution. The resorptive areas or “pits” were imaged using reflective light microscopy. Pit area (µm^2^) per low power field was quantitated using Image Pro Express software. Six fields per experimental condition were evaluated.

### High content image assay

As described above, murine preosteoclasts were obtained *in vitro* from BMMNCs isolated from 6- to 8-week-old WT and *Nf1^+/−^* mice. After being cultured in α-MEM supplemented with M-CSF (30 ng/mL) and RANKL (20 ng/mL), preosteoclasts were starved in 0.5%BSA in α-MEM for 6 hours, pretreated with or without PLX3397 in serial dilution for 2 hours and then stimulated with M-CSF (30 ng/mL) for 5 minutes. The cells were then fixed and incubated with anti-phospho-c-Fms antibody (Cell signaling) at 1∶100 for immunofluorescence staining. Thereafter, the cells were incubated with Alexa Fluor® 488 goat anti-rabbit IgG (H+L) (Invitrogen) at 1∶500 and Hoechst (Invitrogen) at 1∶1000 in PBS at room temperature for 90 minutes and imaged on a BD AttoVision^TM^ instrument. The mean fluorescence intensity of phospho-c-Fms was acquired and normalized to each cell based on the nuclear counts. Data readout was analyzed as phospho-c-Fms mean fluorescence intensity. The half maximal inhibition concentration (IC_50_) of PLX3397 was calculated by Prism software.

### Ovariectomy surgery

To determine if application of PLX3397 prevents bone loss in *Nf1^+/−^*-OVX mice *in vivo*, two groups of 8 week-old WT or *Nf1^+/−^* female mice were subject to ovariectomy surgery (OVX) to induce low bone mass as previously reported [18]. Briefly, the mice were anesthetized via inhalation of 1.5% isoflurane (IsoFlo; Abbott Laboratories, North Chicago, IL) mixed with O_2_ (1.5 liter/minute). Ovariectomy was preceded by a 2 cm midline dorsal skin incision. The peritoneal cavity was incised to access the ovaries, which were then excised with scissors. The success of OVX was verified by examining the uterine weight of the mice following sacrifice (Figure S1A–B).

### Drug administration

After two weeks of recovery from the OVX surgery, the mice were treated with either the PBS vehicle or PLX3397 (80 mg/kg/day) by daily gavage as recommended by the Plexxikon Inc. for a period of twelve weeks. At the end of the treatment, the mice were euthanized for analysis. To ensure that these mice received sufficient amounts of PLX3397, blood was collected in heparin coated tubes by cardiac puncture and plasma was separated to determine the concentration of PLX3397 in the blood (Figure S1C).

### Histomorphometric analysis

Utilizing our previously established methods [18], [24], [25], the femurs of syngeneic experimental mice were fixed with 4% buffered paraformaldehyde (PFA) and decalcified for paraffin embedding. Histological sections from the distal metaphysis were stained for the osteoclast enzyme TRACP. The area of TRACP positive (TRACP^+^) osteoclasts per unit trabecular surface was counted under the microscope with a QImaging camera and QCapture-Pro software (Version 5.1, Fryer Company Inc., Cincinnati, OH).

### Detection of C-terminal telopeptide of type I collagen (CTX) in plasma

To evaluate the impact of PLX3397 on osteoclast activity *in vivo*, the CTX levels in the plasma were measured. Briefly, blood was collected by heart puncture and transferred into ethylenediaminetetraacetic acid (EDTA) coated tubes. The tubes were immediately placed on ice, and centrifuged within 30 minutes for plasma separation. All samples were analyzed in the same assay. The concentration of CTX was determined by using a commercial enzyme immunoassay kit (Ratlaps^TM^ EIA, Immunodiagnostic systems Inc., Fountain Hills, AZ).

### Liquid chromatography/mass spectroscopy (LC/MS) analysis of mice plasma

Plasma was collected following centrifugation at 3,000 rpm at 4°C for 20 minutes and processed for liquid chromatography/mass spectroscopy (LC/MS) analysis. Briefly, murine plasma samples were prepared for LC/MS analysis by precipitating plasma with 3x volumes of ice cold acetonitrile. The precipitated samples were centrifuged and the clarified samples were diluted with 5×0.2% formic acid in water. Samples were analyzed by LC/MS in a formic acid, water, methanol chromatography system using an MDS SCIEX API 3000 spectrometer (Applied Biosystems, Carlsbad, California).

### Measurement of BMD *in vivo*

Bone mineral density (BMD) of the femur was evaluated *in vivo* using peripheral dual energy x-ray absorptiometry (pDEXA) designed for analyzing mouse skeletons (GE-Lunar Corp., Madison, USA). The mice were anesthetized via inhalation of 1.5% isoflurane (IsoFlo; Abbott Laboratories, North Chicago, IL) mixed with O2 (1.5 liter/minute) and were placed in the prone position on a specimen tray for scanning. For this experiment, 8 week-old female mice were scanned for BMD once before surgery. Then, after 12 weeks of drug treatment, the mice were scanned for BMD again before euthanasia. The BMD of the left femoral metaphysis was measured by defining a region of interest of 11 pixels x 10 pixels proximal to the distal growth plate, which contains a high content of trabecular bone.

### Micro (µ) computed tomography (CT)

After euthanasia, bone volume and microarchitecture of the femoral distal metaphysis were evaluated using a desktop µCT (µCT 20; Scanco Medical AG, Bassersdorf, Switzerland). Each specimen was scanned with a slice increment of 9 µm starting at 15% of the total femur length measured from the tip of femoral condyle and extending proximally for 200 slices. Microarchitecture parameters including bone volume (BV, mm^3^) and bone volume fraction (BV/TV, %) were measured.

### Western blotting analysis

The phosphorylation levels of c-Fms, Erk1/2 and p90RSK in preosteoclasts were determined by western blot using phospho-specific antibodies (Cell Signaling, Danvers, MA). Phosphorylation levels were quantitated with Image J and standardized by total ß-actin expression levels (Cell Signaling). Briefly, preosteoclasts were deprived of growth factors for 12 hours in α-MEM supplemented with 0.5% bovine serum albumin (BSA) before being pretreated with PLX3397 at different concentrations as indicated, and were stimulated with M-CSF (30 ng/mL) for 5 minutes in the presence of PLX3397. The cells were then lysed in ProteoJET lysis buffer (Fermentas, Glen Burnie, MD) supplemented with Complete Mini protease inhibitor cocktail (Roche, Indianapolis, IN) for protein extraction. Protein separated by sodium dodecyl sulfate polyacrylamide gel electrophoresis (SDS-PAGE) were transferred to nitrocellulose filters and probed with specific antibodies.

### Statistical analyses

The data are presented as mean ± standard error of the mean (SEM). Differences among experimental groups were analyzed by repeated measures of paired *t-*tests or analysis of variance (ANOVA) with appropriate post hoc *t-*tests. *P* values less than 0.05 were considered significant. Statistical analyses were performed with Prism 5.0 software (GraphPad, La Jolla, CA).

## Results

### Aberrant c-Fms activation mediates M-CSF hyperresponsiveness in *Nf1* haploinsufficient osteoclast progenitors

**Figure 1:**
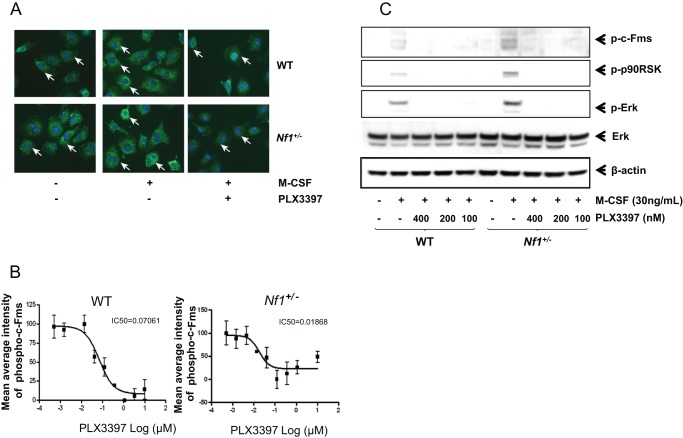
Increased c-Fms activation in *Nf1^+/−^* osteoclast progenitors. (**A**) Expression level of phospho-c-Fms in WT and *Nf1^+/−^* preosteoclasts was evaluated by immunofluorescence staining. Preosteoclasts were obtained by 3 days culture in the presence of M-CSF (30 ng/mL) and RANKL (20 ng/mL). After serum deprivation in 0.5% BSA in α-MEM for 6 hours, cells were pretreated with PLX3397 or PBS vehicle before stimulated with M-CSF (30 ng/mL). The cells were then fixed and stained for phospho-c-Fms (Arrows indicate the phospho-c-Fms positive cells). (**B**) *Nf1^+/−^* osteoclast progenitors show enhanced phosphorylation of c-Fms both at baseline and in the presence of M-CSF. Treatment with PLX3397 significantly reduced the activation of c-Fms both in the WT and *Nf1^+/−^* preosteoclasts. Maximal inhibitory concentration (IC_50_) indicates the half maximal (50%) inhibitory concentration (IC) of a substance (50% IC, or IC_50_). (**C**) After being stimulated with M-CSF (30 ng/mL) for 5 minutes, the phosphorylation levels of c-Fms, Erk, and P90RSK in both WT and *Nf1^+/−^* preosteoclasts were detected by western blot. Pretreatment of PLX3397 completely blocked the activation of Erk and p90RSK in those cells. These experiments were repeated on three independent occasions with similar results.

Although *Nf1* ^+/−^ osteoclasts and osteoclast progenitors are hyperresponsive to limiting doses of M-CSF, the molecular mechanisms mediating this phenotype remain unclear. We therefore sought to determine whether *Nf1* haploinsufficiency may result in aberrant M-CSF signaling via hyperactivation of its receptor, c-Fms. We first examined the phosphorylation level of c-Fms by immunofluorescence staining of WT and *Nf1^+/−^* preosteoclasts. *Nf1^+/−^* preosteoclasts exhibited increased phospho-c-Fms staining both at base line and after being stimulated with M-CSF (30 ng/mL) for 5 minutes as compared with WT preosteoclasts. The expression levels of phospho-c-Fms decreased markedly following treatment with a small molecule inhibitor of the c-Fms receptor tyrosine kinase, PLX3397, at 62.5 nM and reached the lowest level at 1 µM in WT cultures and 0.1 µM in *Nf1^+/−^* cultures, respectively (Figure 1A and 1B).

To further validate these findings and determine the impact of increased c-Fms activation with respect to the downstream MAPK pathway, the phosphorylation levels of c-Fms, p90RSK, and Erk were detected by western blotting. *Nf1^+/−^* preosteoclasts demonstrated increased phosphorylation levels of c-Fms, p90RSK and Erk as compared with WT preosteoclasts when stimulated by M-CSF. Pretreatment of PLX3397 blocked the phosphorylation of these proteins both in WT and *Nf1^+/−^* preosteoclasts (Figure 1C and Figure S2).

#### Pharmacologic c-Fms inhibition reduces osteoclast progenitor frequency and osteoclast differentiation *in vitro*

**Figure 2:**
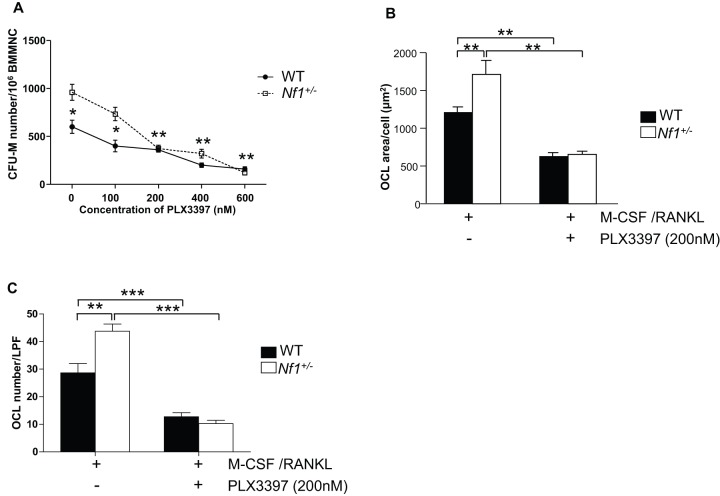
c-Fms hyperactivity mediates increased osteoclast progenitor frequency and differentiation capacity in bone marrow cells from *Nf1^+/−^* mice. (**A**) Clonogenic assays were performed to establish the number of osteoclast progenitors, CFU-M, per femur from WT and *Nf1^+/−^* mice. Addition of PLX3397 reduced CFU-M at a dose dependent manner. *p<0.05 and **p<0.01. (**B**) The ability of WT and *Nf1^+/−^* BMMNCs to form osteoclasts in response to M-CSF with PLX3397 or PBS vehicle was evaluated by TRACP staining. The y-axis shows the osteoclast area per cell. (**C**) The numbers of TRACP^+^ osteoclasts in WT and *Nf1^+/−^* culture was evaluated by TRACP staining. Data are represented as mean ± SEM, with n = 3. **p<0.01 and ***p<0.001 compared with the control group. Three independent experiments were conducted with similar results.

Given that *Nf1* haploinsufficiency leads to M-CSF induced hyperactivation of c-Fms and the downstream MAPK effector pathway, we sought to investigate the effect of pharmacologic c-Fms inhibition on *Nf1^+/−^* osteoclast differentiation *in vitro*. Since osteoclasts are derived from monocyte/macrophage progeny within the bone marrow, we quantified the number of colony forming unit-macrophage (CFU-M) per femur following culture in semisolid methylcellulose media. Consistent with our previous data [18], a significantly increased number of CFU-M was observed in *Nf1^+/−^* bone marrow mononuclear cells (BMMNCs) as compared to WT BMMNCs. Addition of PLX3397 significantly reduced the frequencies of CFU-M at concentrations of 200 nM both in WT and *Nf1^+/−^* cultures (Figure 2A).

To assess whether inhibition of M-CSF signaling affected osteoclast formation, we established osteoclast cultures in the presence of M-CSF/RANKL with PBS vehicle or PLX3397. Osteoclast differentiation was determined by calculating the TRACP^+^ area per osteoclast (Figure 2B) and by counting the number of TRACP^+^ osteoclasts per low power field (Figure 2C). *Nf1^+/−^* cultures contained significantly increased TRACP^+^ area and osteoclast numbers as compared to WT cultures Addition of PLX3397 reduced osteoclast formation in both WT and *Nf1^+/−^* cultures.

### M-CSF/c-Fms signaling mediates *Nf1^+/−^* preosteoclast migration and adhesion

**Figure 3:**
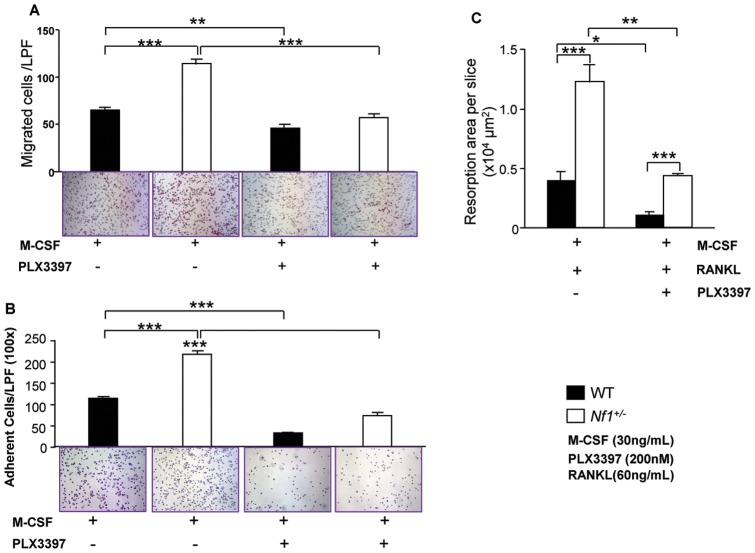
Pharmacologic c-Fms inhibition mitigates multiple *Nf1^+/−^* osteoclast gain in functions including migration, adhesion, and bone resorptive activity. (**A**) A transwell assay was used to investigate the effect of the c-Fms inhibitor PLX3397 on osteoclast migration. Experiments were conducted in triplicates per condition. Five fields were counted per well. Three independent experiments were performed with similar results. **p<0.01;. (**B**) Adhesion assays were used to investigate the effect of the PLX3397 on osteoclast attachment to vitronectin. Experiments were conducted in triplicates per condition. Five fields were counted per well. Three independent experiments were performed with similar results. ***p<0.001. (**C**) Quantitative evaluation of bone resorption. Data represent the mean ± SEM of 6 fields per genotype. Each condition was carried out in triplicates and four independent experiments were performed. *p<0.05; **p<0.01; ***p<0.001.

The ability of osteoclasts to migrate across the bone surface is a key cellular function required for bone resorption. We have previously reported that haploinsufficiency of *Nf1* resulted in enhanced preosteoclast migration in response to M-CSF [18]. To assess the impact of c-Fms inhibition on preosteoclast motility mediated by M-CSF, preosteoclast migration was assessed by transwell assays. Bottom chambers containing media alone without M-CSF were used as negative controls (data not shown). After four hours of stimulation, M-CSF induced a significantly higher level of migration in *Nf1^+/−^* cultures than that of WT cultures (Figure 3A). Addition of PLX3397 reduced *Nf1^+/−^* preosteoclast migration to WT levels.

To determine the impact of M-CSF/c-Fms signaling on preosteoclast adhesion, a key process for initiation of bone resorption, adhesion assays were performed. Consistent with our previous findings [18], significantly increased *Nf1^+/−^* preosteoclasts were observed to adhere to vitronectin following M-CSF stimulation. Pretreatment with PLX3397 (200 nM) dramatically decreased the number of adherent cells in both WT and *Nf1^+/−^* preosteoclasts mediated by M-CSF (Figure 3B).

### PLX3397 inhibits osteoclast bone resorption

To assess the impact of PLX3397 on osteoclast bone resorptive activity, pit formation assays were performed [18], [19]. Consistent with our previous study [18], *Nf1^+/−^* osteoclasts induced a 2–3 fold increased pit forming area as compared to WT osteoclasts (Figure 3C). Importantly, PLX3397 reduced pit forming areas in both WT and *Nf1^+/−^* cultures. These data indicate that c-Fms inhibition not only inhibits osteoclast differentiation but also diminishes osteoclast bone resorptive activity.

### Pharmacologic c-Fms inhibition prevents bone loss in ovariectomized *Nf1^+/−^* mice

Based on our findings that *Nf1^+/−^* osteoclasts exhibit increased bone lytic activity and that PLX3397 inhibits osteoclast formation and bone resorptive activity *in vitro*, we postulated that pharmacologic inhibition of c-Fms with PLX3397 may exert anti-catabolic effects on bone *in vivo*. To further test our hypothesis that PLX3397 may prevent bone loss *in vivo,* we gavage-fed PLX3397 to *Nf1^+/−^* mice undergoing ovariectomy (OVX), an established osteoporotic stress model [18]. 80 mg/kg/day of PLX3397 were administered to both WT-OVX and *Nf1^+/−^*-OVX mice for a period of twelve weeks, and PBS was used as vehicle in control groups.

**Figure 4:**
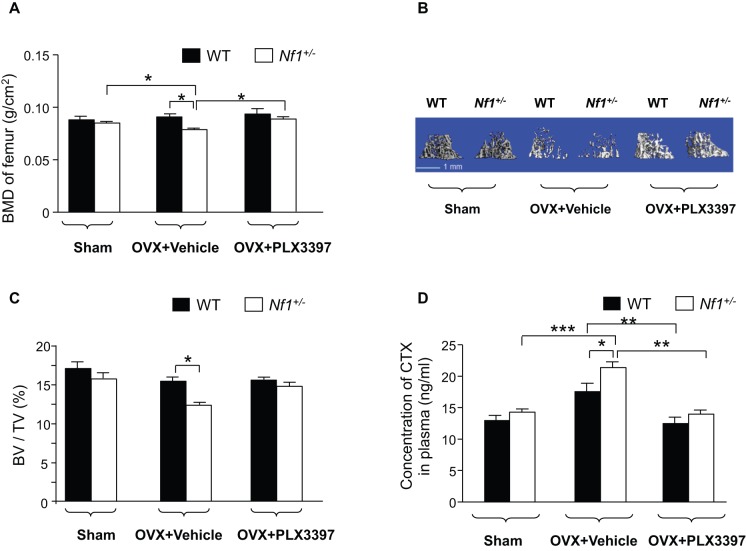
PLX3397 treatment restores bone mass in *Nf1^+/−^* OVX mice *in vivo*. (**A**) The BMD of femoral metaphysis was measured by pDEXA and analyzed with Lunar Piximus. *p<0.05; ***p<0.001 (**B**) Micro-CT (μCT) scans were performed on the distal femur of all groups of mice. Representative reconstructed 3D pictures are shown. The cortical portion of each femur has been removed to allow visualization of the metaphyseal architecture. (**C**) Bone volume fraction (BV/TV) was assessed by μCT. *p<0.05. (**D**) The concentration of CTX in plasma in WT and *Nf1^+/−^* mice was measured after OVX. Data are represented as mean ± SEM, with n = 8. *p<0.05, **p<0.01 and ***p<0.001 compared with the control group.

Similar to our previous study, the OVX surgery resulted in reduced BMD in both genotypes of mice [18]. Moreover, the *Nf1^+/−^* -OVX mice lost significantly more bone mass than the WT- OVX mice (Figure 4A), confirming that a pro-resorptive challenge induces a greater osteoclastic response in *Nf1* haploinsufficient mice. *Nf1^+/−^*-OVX mice fed with PLX3397 showed a significantly increased BMD as compared to the vehicle control group.

To further investigate the role of PLX3397 on modulating trabecular bone, microcomputed tomography (μCT) was performed to examine the architecture of trabecular bone. Representative μCT 3D reconstructions are shown (Figure 4B). *Nf1^+/−^*-OVX mice displayed significantly less trabecular bone, as determined by bone volume/tissue volume (BV/TV), and as compared to WT-OVX mice that received vehicle treatment. PLX3397 significantly increased the trabecular bone in *Nf1^+/−^*-OVX mice (Figure 4C).

In addition, CTX, a sensitive marker of bone loss, was significantly increased in the plasma of both WT-OVX and *Nf1^+/−^*-OVX mice as compared to controls (*p<0.05 for *Nf1^+/−^*-OVX vs. *Nf1^+/−^* sham) (Figure 4D). CTX was even higher in the plasma of *Nf1^+/−^*-OVX mice than that of WT-OVX mice (*p<0.05 for *Nf1^+/^* ^−^-OVX vs. WT-OVX). Importantly, administration of PLX3397 attenuated the plasma CTX levels in both WT-OVX mice and *Nf1^+/−^*-OVX mice. Collectively, these data suggest that pharmacologic c-Fms inhibition is sufficient to prevent bone loss by abrogating osteoclast bone resorptive activity *in vivo*.

### c-Fms hyperactivity mediates expansion of the osteoclast progenitor pool in *Nf1^+/−^* OVX mice *in vivo*

**Figure 5:**
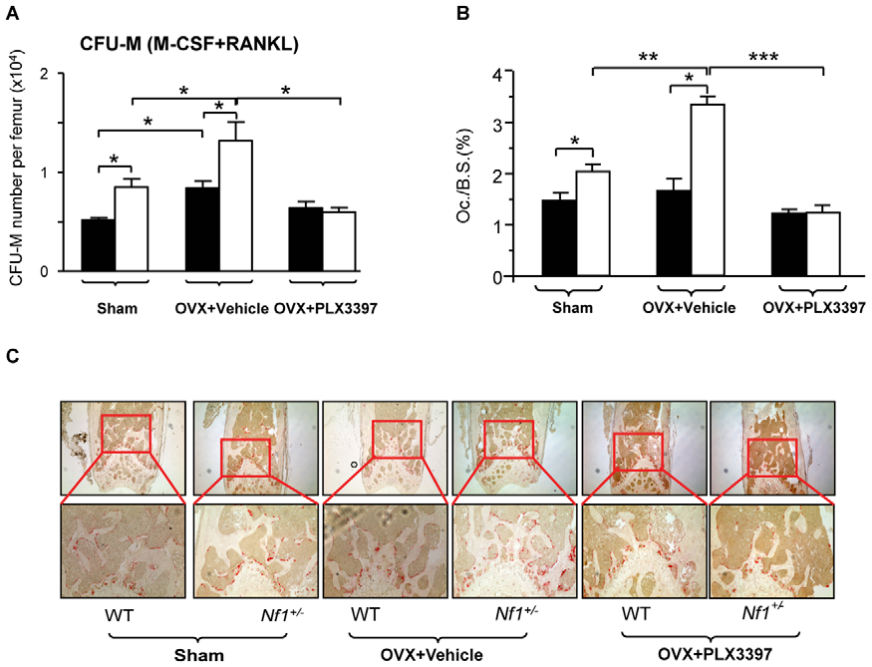
c-Fms inhibition attenuates osteoclast formation *in vivo*. (**A**) The CFU-M number of the BMMNC from WT and *Nf1^+/−^* mice after OVX with vehicle or PLX3397 treatment was counted. *p<0.05. (**B**) Quantitative analysis of the percentage of TRACP^+^ osteoclast area/bone surface area (Oc./BS). Data are represented as mean ± SEM, with n = 5. *p<0.05; **p<0.01; ***p<0.001. (**C**) Femur samples were processed for TRACP staining. Representative photographs (40x upper panels, 100x lower panels) of the trabecular bone following TRACP staining are shown. The red stained area indicates TRACP^+^ osteoclasts.

Since osteoclasts are considered to be the key lineage mediating bone loss in ovariectomy induced resorptive stress, we next investigated the effect of ovariectomy on the number of osteoclast progenitors in the bone marrow by performing CFU-M assays. Ovariectomy surgery significantly increased the frequency of CFU-M in both WT-OVX and *Nf1^+/−^*-OVX groups as compared to the sham group. PLX3397, but not by vehicle gavage-feeding, suppressed the CFU-M formation to the level of sham groups in the *Nf1^+/−^* mice (Figure 5A).

To examine the impact of PLX3397 on osteoclastogenesis *in vivo*, the TRACP^+^ cell area/bone surface area of the femur was scored following TRACP staining. A significant increase in the TRACP^+^ area/bone surface area was observed in *Nf1^+/−^* Sham as compared to the WT Sham group. Furthermore, OVX surgery induced an even more dramatic increase in osteoclastogenesis in *Nf1^+/−^* mice as evidence by the increased TRACP^+^ cells compared to WT-OVX mice. Importantly, PLX3397 treatment reduced the number of TRACP^+^ osteoclasts in *Nf1^+/−^_-_*OVX mice (Figure 5B and 5C). Collectively, these data indicate that elevated c-Fms signaling, following ovariectomy induced resorptive stress, results in increased numbers of osteoclast progenitors and mature osteoclasts in *Nf1^+/−^* mice, and consequently accelerated bone loss.

## Discussion

Osteoporosis and osteopenia affect both children and adults with NF1 [5], [6], [7], [12], [26], [27]. As *Nf1^+/−^* mice have normal bone mass, ovariectomy is an established experimental model to induce low bone mass in Nf1+/− mice [18]. Previously, we reported an increased frequency of osteoclast progenitors in *Nf1^+/−^* bone marrow induced by M-CSF as assessed by the colony forming unit-macrophage (CFU-M) assay [18]. We also showed that haploinsufficiency of *Nf1* results in hypersensitivity of osteoclast progenitors to M-CSF correlated with hyperactivation of the Ras/MAPK pathway [18]. In the present study, we show that the M-CSF receptor, c-Fms, is hyperactivated in *Nf1* haploinsufficient osteoclasts and progenitors following M-CSF stimulation. Utilizing a small molecule c-Fms kinase inhibitor, PLX3397, we demonstrate that c-Fms overactivity mediates multiple *Nf1^+/−^* osteoclast gain-in-function phenotypes both *in vitro* and *in vivo* including expansion of osteoclast progenitors in the bone marrow, increased osteoclast migration, adhesion, and differentiation, and accelerated bone loss following ovariectomy induced resorptive stress.

M-CSF is a critical cytokine for the development of macrophages and their subsequent differentiation to the osteoclast cell lineage [28]. It has been reported recently that macrophage colony stimulating factor (M-CSF), together with signals from a family of biologically related tumor necrosis factor receptor like proteins (particularly RANKL), control the induction of osteoclast differentiation and are key to the physiologic and pathologic responses of osteoclasts [29], [30]. Post-menopausal patients, as well as those with common chronic diseases such as arthritis, have a high incidence of osteoporosis, due to increased production of M-CSF and RANKL, which leads to increased osteoclast activation and consequent bone loss [31]. It has been shown that PI3K is a critical downstream effector of the M-CSF/c-Fms pathway [32]. Once activated by M-CSF, PI3K modulates osteoclast survival as well as actin remodeling [33], [34]. Pharmacologic inhibitors of PI3K, including LY294002 and wortmannin, show a dramatic reduction of osteoclast formation in cultures treated with M-CSF and RANKL. Furthermore, treating osteoclasts with pharmacologic inhibitors of PI3K also impairs the resorptive activity of osteoclasts, which is associated with impaired membrane ruffling, deficient actin ring formation, and reduced pit formation [35]. Unfortunately, the clinical utility of commercially available PI3K inhibitors such as wortmannin and LY294002 is limited as these compounds target the ATP-binding site of all PI3K family members and interfere with other PI3K related kinases at therapeutic concentrations. As such, these inhibitors are broad spectrum, non-specific and are associated with significant toxicity *in vivo*. PLX3397 is a novel small molecule that potently and selectively inhibits c-Fms, Kit, and Flt3-ITD kinases [36], [37]. Since osteoclasts cultured from *Nf1^+/−^* mice and human NF1 patients exhibit hyperactivation of the c-Fms receptor kinase in response to M-CSF, we reasoned that pharmacologic inhibition of c-Fms with PLX3397 may exert anti-catabolic effects on bone in the NF1 murine model.

M-CSF is critical for the proliferation of osteoclast precursors and addition of an M-CSF neutralizing antibody considerably reduced macrophage differentiation at the early precursor stage [38], [39]. Consistent with these findings, our *in vivo* study indicates that administration of PLX3397 significantly reduced the frequency of osteoclast progenitors as shown by reduced numbers of CFU-M. In addition, M-CSF is also essential for osteoclast formation and maturation by contributing to proliferation and survival [1√8] [40]. In the present study, we show that pharmacologic c-Fms inhibition reduces osteoclast differentiation and bone resorption. In addition, our data demonstrates that PLX3397 inhibited M-CSF mediated MAPK signaling pathway by diminishing the activation of c-Fms, p90RSK and Erk, which are key to the hyperfunctioning of *Nf1^+/−^* osteoclasts in response to M-CSF.

Underscoring the critical role of c-Fms in mediating excessive osteoclast bone resorptive activity in NF1, administration of PLX3397 was sufficient to prevent bone loss in an established murine model of NF1 low bone mass. This normalization of bone mineral density and trabecular bone mass in response to pharmacologic c-Fms inhibition *in vivo* was associated with the attenuation of osteoclast development and cellular functions, including osteoclast differentiation, migration and bone resorption. Collectively, these data suggest that the M-CSF signaling through its receptor c-Fms may play a critical role in osteoclast-mediated osteoporotic manifestations in NF1. Biochemically, our data show that administration of PLX3397 can prevent the activation of the MAPK signaling cascade in response to M-CSF, thereby correcting aberrant osteoclast functions in the *Nf1^+/−^* murine model. As such, further studies are needed to assess whether targeting M-CSF/c-Fms signaling might provide a potential treatment for osteoporosis in NF1.

## Supporting Information

Figure S1 **(A)** Uteri of WT or *Nf1^+/−^* mice undergoing OVX surgery were compared to sham operated controls. **(B)** Uterine weight of WT and *Nf1^+/−^* mice undergoing OVX surgery vs. sham controls. Data are represented as mean ± SEM, with n = 5. *p<0.05; **p<0.01; ***p<0.001. (**C**) The plasma concentration of PLX3397 in mice receiving treatment is shown.
(EPS)

Figure S2 **The phosphorylation levels of c-Fms, Erk, and p90RSK in both WT and** ***Nf1+/−*** **preosteoclasts were detected by western blot (** **Figure 1C** **) and quantified by densitometry as shown below.**
(EPS)
